# Rotavirus-specific neutralization assay enables evaluation of mucosal immune responses

**DOI:** 10.3389/fimmu.2025.1677823

**Published:** 2025-12-02

**Authors:** Maxi Harzer, Antje Rückner, Thomas W. Vahlenkamp

**Affiliations:** Institute of Virology, Faculty of Veterinary Medicine, University of Leipzig, Leipzig, Germany

**Keywords:** virus neutralization assay, rotavirus, swine, mucosal immunity, saliva

## Abstract

Mucosal infections are a major global health concern in humans and animals. Neutralization assays are considered the gold standard for evaluating functional antibodies but are typically limited to serum analysis. This often excludes mucosal sample types such as saliva or intestinal mucus, which may better reflect local immunity, particularly for enteric pathogens such as Rotaviruses (RV). We optimized and standardized a virus neutralization assay (VNA) for RVA to evaluate its applicability across serum, intestinal mucus, and saliva samples. Five key protocol variables were systematically assessed: (i) the applied trypsin concentration, (ii) the incubation time for proteolytic activation of the RV, (iii) the infection dose applied, (iv) the duration of neutralization time and (v) sample-specific factors that affect assay performance. The following parameters have proven to be optimal: (i) end concentration of 20 µg/ml Trypsin (ii) two hours for proteolytic activation of the RV, (iii) MOI of 0.5 – 3.0 x 10^-3^, (iv) two or eight hours of neutralization time for serum or saliva and mucus samples. Optimization of trypsin concentration and virus activation time significantly improved assay reproducibility across sample types. Analysis of the three validated sample materials of individual swine demonstrate that RV-specific neutralizing antibodies can be reliably detected in saliva. Saliva-based neutralization titers strongly correlated with those from intestinal mucus, indicating that salivary antibodies may serve as a non-invasive surrogate for gut mucosal immunity. The optimized VNA enables robust detection of mucosal neutralizing antibodies in saliva and intestinal mucus, offering a standardized, scalable approach for evaluating mucosal immune responses following RV infection or vaccination. This platform holds promise for broader application in mucosal immunology and vaccine monitoring, although its applicability in low-resource and pediatric settings still needs to be demonstrated.

## 1 Introduction

For regulatory approval of vaccines, demonstrating protection against infection and/or clinical symptoms is a fundamental requirement. The SARS-CoV-2 pandemic has highlighted the critical importance of identifying robust and meaningful correlates of protection against infection or disease (1–3). Most pathogens enter the body via mucosal surfaces; therefore, immune defense at these entry points is most effective in preventing infection (4, 5). The induction of local immunity is thus a primary objective of prophylactic vaccination strategies (6).

A direct correlate of protection against mucosal associated pathogens is local immunity mediated by humoral and cellular components of the mucosal immune system. Local antibodies in the gastrointestinal tract have been identified in several studies as direct correlates of protection (7–10). According to the definition by Plotkin and Gilbert 2012 (11), these antibodies can be considered mechanistic correlates of protection against enteric diseases.

However, sampling small intestinal secretions requires invasive procedures that are unsuitable for routine diagnostics. Therefore, other correlates are needed to infer local immune status. Mucosal immune responses are not limited to the site of antigen exposure but also occur at distant mucosal effector sites (12). Based on this principle, saliva has been investigated as a surrogate for downstream mucosal responses within the enteric system (13).

Saliva is increasingly used in both human and veterinary medicine for direct and indirect pathogen detection (14–17). However, standardizing oral fluid–based antibody diagnostics remains challenging. Variability in sampling methods (15, 18, 19), test protocols (18, 20), and individual physiological factors can significantly influence antibody concentrations and introduce high variability (21–23). Furthermore, antibody titers in saliva are typically lower than those in serum (24). Consequently, the successful application of saliva-based diagnostics depends on the use of highly sensitive and robust assay systems.

Among the various antibody detection methods, the determination of neutralizing antibodies is particularly important for evaluating infection events and vaccine efficacy. However, suitable and standardized virus neutralization assays (VNAs) are lacking for most pathogens (25), as assay performance is influenced by both pathogen-specific properties and the nature of the sample material (26–29).

In this study, we validated a rotavirus-specific VNA for quantifying antibody titers in saliva, serum, and intestinal mucus in the pig model. Neutralizing antibodies target epitopes on two viral surface proteins, VP4 and VP7. Rotaviruses (RVs) are non-enveloped viruses of the family *Reoviridae*, characterized by a triple-layered capsid. They are mucosal associated pathogens that infect and replicate in mature enterocytes, causing gastrointestinal diarrheal disease in mammals, birds, and reptiles.

The introduction of rotavirus vaccines in both humans and animals has significantly improved global health by reducing rotavirus-associated mortality. However, current vaccines remain less effective in protecting children in low-income countries. Several factors have been proposed to explain the underperformance of oral vaccines (30, 31). However, in the absence of a robust correlate of protection, efforts to improve vaccine efficacy or to optimize environmental conditions remain challenging. Saliva-based investigations may offer a promising, noninvasive, and practical diagnostic tool for evaluating vaccine- and infection-induced mucosal immune responses in the gastrointestinal tract.^1^.

## 2 Materials and equipment

All required materials and reagents are listed in **Table 1**. All required equipment and instruments are listed in **Table 2**.

**Table 1:** Materials and reagents.

Reagent/instrument	Supplier/source	Specification/concentration
DMEM Medium	Gibco (#41965)	1×
Acetone	Carl Roth (#9372.2)	80%
Bovine Serum Albumin (BSA)	Sigma-Aldrich (#A4503)	1%
Dulbecco’s Phosphate-Buffered Saline (DPBS)	Gibco, 10× without Ca^2+^/Mg^2+^	1×
Fetal Calf Serum (FCS)	Sigma-Aldrich	—
Trypsin/EDTA	Life Technologies	10 µg/mL, 20 µg/mL
Penicillin/Streptomycin	Gibco, 10,000 U/mL, 200×	1×
Rotavirus A strain Pig-tcGER2018Over G9P[32]	Field isolate	—
Rotavirus A strain Lincoln G6P[1]6	Zoetis Deutschland GmbH	—
Rotavirus A strain OSU G5P[7]9	Institute of Virology, Faculty of Veterinary Medicine, University of Leipzig	—
Anti-RVA VP6 primary antibody (rabbit)	Custom antibody (TV 14/12)	1:2000
Anti-RVC VP6 primary antibody (rabbit)	Custom antibody (TV 14/12)	1:4000
Goat anti-rabbit IgG (H+L) Alexa Fluor™ 488 (cross-adsorbed)	Life Technologies (#A-11008)	RRID: AB_143165
Goat anti-Pig IgA Secondary Antibody FITC	Life Technologies (#PA1-84624)	RRID: AB_931397
Goat anti-pig IgG (H+L) DyLight 488	Abcam (#ab102139)	RRID: AB_10710341

**Table 2:** Equipment and instruments.

Device	Model/source	Details/RRID
Imaging system (fluorescence and chemiluminescence)	Fusion FX7, peqLab	—
Electrophoresis chamber	RELSPLUSST25, Roth	—
Analytical balance	PM400, Mettler Toledo	—
Fluorescence microscopes	IX70, Olympus	—
Digital microscope	BZ-X, Keyence	RRID: SCR_018604
Freezer (−20 °C)	Comfort series, Liebherr	—
Freezer (−80 °C)	KM-DU53Y1E, Liebherr/Panasonic	—
CO_2_ Incubator	Model 3.1, Binder	—
Refrigerated storage (4 °C)	Comfort series, Liebherr	—
Magnetic stirrer	RSM-10HP, Phoenix Instruments	—
Neubauer counting chamber	Assistent	—
Glass pipettes	Labsolute	10 mL and 5 mL
Bead mill homogenizer	TissueLyser II, Qiagen	RRID: SCR_018623
Pipetting aid	peqLab	—
Biosafety cabinet	HERAsafe, Heraeus Instruments	—
Medium bottles	Greiner Bio-One	100 mL, 500 mL
Parafilm	Carl Roth (DMSO: C_2_H_6_OS)	—
Pipette tips	Greiner Bio-One	10, 20, 100, 200, and 1000 µL
Polypropylene tubes	Greiner Bio-One	15 mL and 50 mL
Microcentrifuge tubes	Greiner Bio-One	1.5 mL and 2 mL
Saliva collection swabs	Sarstedt Salivette™	—
Serum tubes	Sarstedt S-Monovette # 13395	9ml
Syringes	Ilona Schubert Laboratory Supplies	1 mL, 10 mL, 50 mL
Syringe filters	Ilona Schubert Laboratory Supplies	0.20 µm, 0.45 µm
Biosafety cabinet	Hs 12/2, Heraeus Instruments	—
Cell culture flasks	Greiner Bio-One	25 cm², 75 cm², 175 cm²
Cell culture plates	Greiner Bio-One	96-well
MA104 cell line	ATCC CRL-2378.1	RRID: CVCL_3846

## 3 Methods

### 3.1 Indirect and direct immunofluorescence assay

MA 104 cells grown in 96-well-plates were fixed with 80% acetone for 15 min at 4 °C. For direct IFA, either a polyclonal anti-VP6 RVA serum (rabbit anti RVA-VP6, produced by immunizing rabbits with purified VP6 used at a 1:2000 dilution in PBS containing 1% BSA) or an anti-RVC serum (rabbit anti RVC-VP6, used at a 1:4000 dilution in PBS with 1% BSA) was applied. For indirect IFA, serum samples were diluted as required and applied after blocking with 5% BSA in PBS for 30 minutes at 37 °C. Sixty microliters of the sample were added to each well of a 96-well cell culture plate, followed by 60 min incubation at 37 °C. Subsequently, wells were washed three times with PBS containing 1% BSA. Then, the secondary antibody solution was added and incubated for 30 minutes at 37 °C. The secondary antibodies used included Goat anti-pig IgG (H+L) DyLight 488, Goat anti-Pig IgA FITC for indirect, and Goat anti-rabbit IgG (H+L) Alexa Fluor™ 488 (cross-adsorbed) for direct IFA respectively. All secondary antibodies were diluted in PBS with 1% BSA to a final concentration of 1 µg/ml. DAPI was added to the solution at a final concentration of 0.5 µg/ml. After two additional washes with PBS containing 1% BSA, a final wash with PBS alone was performed. Fluorescence signals were visualized using an inverted fluorescence microscope. In the direct IFA, the presence of any RVA VP6- or RVC VP6-specific fluorescence signal within a well was visually determined and documented. In the indirect IFA, the absence of fluorescence was independently assessed by two investigators to minimize subjective bias and ensure consistency of the evaluation.

### 3.2 Validation of virus neutralization assay

The basic protocol of the VNA included pre-activation of the RV with trypsin. A two-fold serial dilution of serum, saliva or intestinal mucus samples was prepared in a 96-well cell culture plate. Serum samples were pre-diluted 1:10. Intestinal mucus samples were mixed 1:50 with PBS and homogenized using a bead mill at 2000 oscillations/min for 30 s. RV was added to each sample, followed by an adsorption step at 37 °C in a 5% CO2 atmosphere. For intestinal mucus samples, the virus-sample mixture was added to a confluent monolayer of MA104 cells, incubated for two hours, and then washed twice with DMEM. For serum and saliva samples, cultures of MA104 cells were detached by trypsinization, centrifuged at 700 x g to remove trypsin-containing medium, resuspendend in DMEM and subsequently seeded into the virus-sample mixture at a concentration of 0.4 × 10^5^ cells/well. After 24 hours of incubation at 37 °C in a humidified atmosphere containing 5% CO_2_, cells were washed once with PBS, fixed with 80% acetone for 15 minutes at 4 °C, washed once with PBS and subsequently analyzed by IFA.

Several steps within the basic protocol were optimized based on virus- and sample-specific properties. Final protocol is presented in **Supplementary Material 1**.

#### 3.2.1 Concentration- and time-dependent effects of trypsin on RV infectivity

To assess the concentration- and time-dependent effects of trypsin on RV infectivity, confluent MA104 cell layers were infected, fixed, and analyzed via direct IFA. Fluorescent foci were counted in four randomly selected microscopic fields at 20× magnification, and the arithmetic mean was calculated.

#### 3.2.2 Influence of virus–antibody adsorption time

The influence of virus–antibody adsorption time on VNA performance was investigated by testing incubation times of 1, 4, 8, 16, and 24 hours for serum, saliva, and intestinal mucus samples.

#### 3.2.3 Influence of inhibition rate on read out results

In parallel with the evaluation of optimal incubation times, the influence of the inhibition rate on assay results and the standard deviation in repeatability was assessed. Accordingly, inhibition rates of 80%, as described in the basic protocol of Jiang et al. (1999), and 100% were compared. For the determination of 80% inhibition, foci within four microscopic fields (MF) per well were counted, and the average number of foci was calculated and expressed as a percentage relative to the positive control. In addition, saliva samples were analyzed both filtered and unfiltered.

#### 3.2.4 Determination of optimal virus concentration

To determine the optimal virus concentration, VNAs were conducted using various multiplicities of infection (MOIs), including negative controls (one fetal calf serum and one pig serum). The extent of fluorescence was assessed relative to maximum fluorescence. Therefore, foci within four MF per well were counted. The average number of foci was calculated and expressed as a percentage relative to the positive control.

#### 3.2.5 Determination of VNA sensitivity

The sensitivity of the VNA protocol was evaluated by comparing absolute RVA-specific IgG titers in serum as described in 3.1 with the corresponding maximum RVA VNA titers within the sera from vaccinated animals.

#### 3.2.6 Determination of intra- and inter-assay repeatability

Intra- and inter-assay repeatability was assessed using technical duplicates and 10–20 biological replicates of the G5P[7]9 RVA-specific VNA. Samples included one serum each with high, medium, and low titers; one saliva sample each with medium and low titers; and one mucus sample each with high, medium, and low titers.

Titer ratios were calculated following linearization of the underlying exponential titer progression according to the following formula:


$$linearised\;VNA-titer=log_{2}\;VNA-titer$$

### 3.3 Sampling protocol for natural infected animals

**Figure 1:**
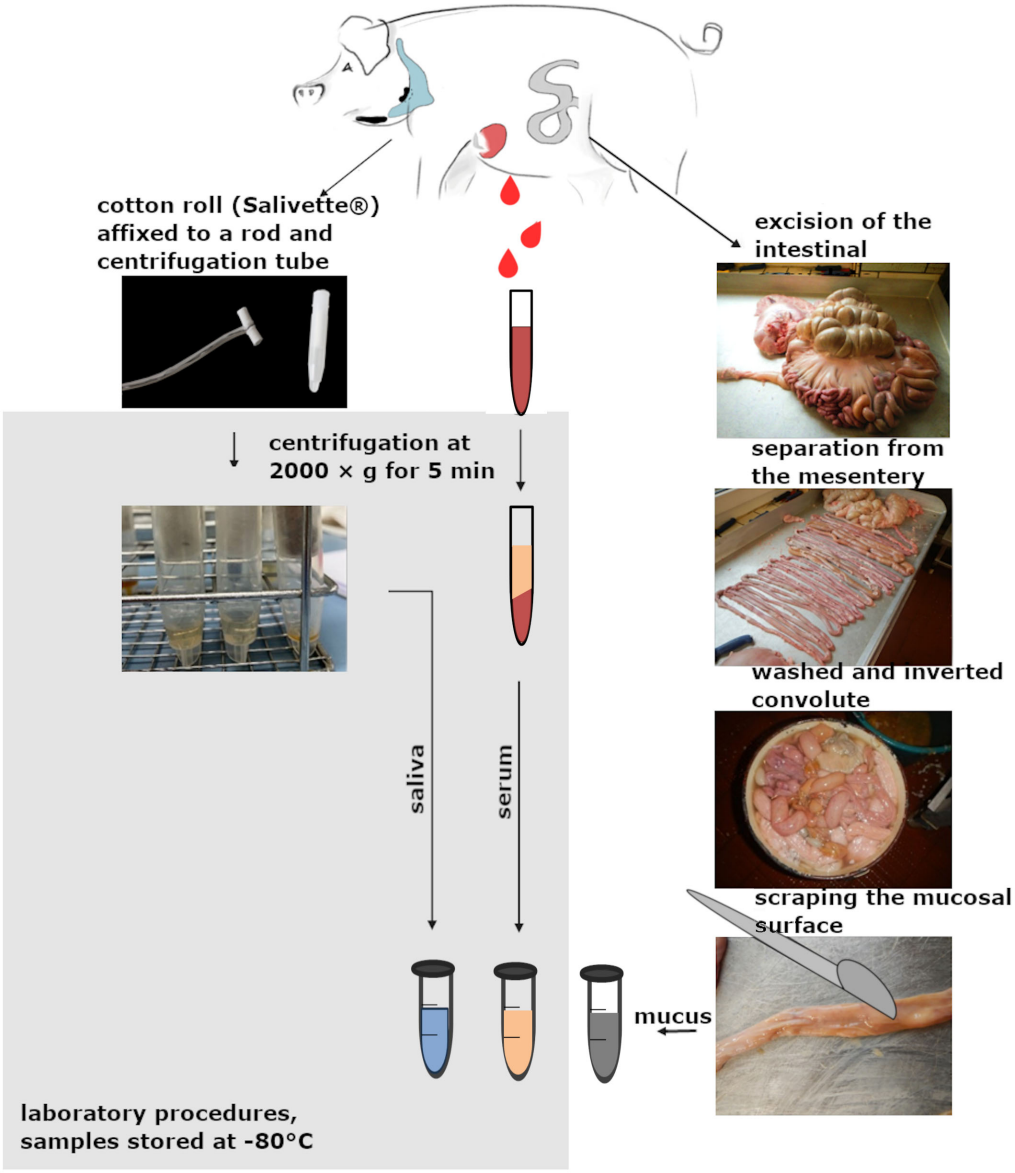
Schematic illustration of sampling. Saliva, serum and intestinal mucus samples were obtained from the duodenum, jejunum and ileum of each pig during domestic slaughter. The saliva was collected using salivettes and centrifuged in the laboratory. Blood from punctured cavernous vein was collected in a tube and centrifuged in the laboratory. Before obtaining the intestinal mucus, the intestine was subjected to several washing steps. All materials obtained were transferred to 2 ml reaction tubes and stored at -80°C. (Created with biorender.com)

Samples were collected from pigs during 16 slaughter sessions (**Figure 1**). Saliva was collected using a cotton roll (Salivette^®^), which was affixed to a rod and offered to the unrestrained animal for oral manipulation. During approximately 20 seconds of interaction and mastication, saliva was absorbed into the cotton roll.

Blood samples were obtained by incising the large blood vessels near the heart. Blood was collected into serum tubes approximately 5 cm downstream from the incision site, directly from the central blood flow.

Following excision of the intestinal convolute, the intestine was bluntly separated from the mesentery. The intestinal contents were then flushed out using hand-warm water. The intestinal lumen was everted, and the intestines were washed again with hand-warm water. Mucus samples were collected by scraping the mucosal surface with the blunt edge of a scalpel at three defined locations: approximately 10 cm distal to the gastric outlet (duodenum), midway along the small intestine (jejunum), and approximately 10 cm proximal to the ileocecal junction (ileum). All samples were transferred into sterile 2 ml reaction tubes.

Samples collected during slaughter were stored at 4 °C until further processing. Within 24 hours, serum was separated in the laboratory, and saliva was extracted from the cotton rolls by centrifugation at 2000 × g for 5 minutes. Prior to storage, up to 700 µl of saliva was filtered through a sterile 0.4 µm pore-size filter. Serum, saliva, feces, and intestinal mucus samples from each individual animal were stored at –80 °C until further analysis.

### 3.4 Sampling protocol for vaccinated animals

In February 2019, a herd-specific rotavirus A (RVA) vaccine was implemented as part of the health management program in a piglet-producing herd. In accordance with the recommendations of the Standing Veterinary Vaccination Committee (STIKO Vet) (32), the vaccine was initially administered to a pilot group of thirty newly acquired gilts.

Four vaccine doses were administered to the gilts, each dose containing 0.6 × 10^6^ focus-forming units (FFU) of an RVA strain of genotype G9P[32]x. The vaccine was administered subcutaneously at the base of the ear at four-week intervals, starting two weeks after arrival. Gilts were seven months of age at the time of the first vaccination. Booster vaccinations were given four weeks (week 18 post-initial vaccination) and two weeks (week 20 post-initial vaccination) prior to farrowing.

Whole blood and saliva samples were collected from eight animals at weeks 0, 2, and 4, as well as at weeks 18 and 22 post-initial vaccination. Serum and saliva samples were stored at –80°C until further analysis.

## 4 Results

### 4.1 VNA validation

**Figure 2:**
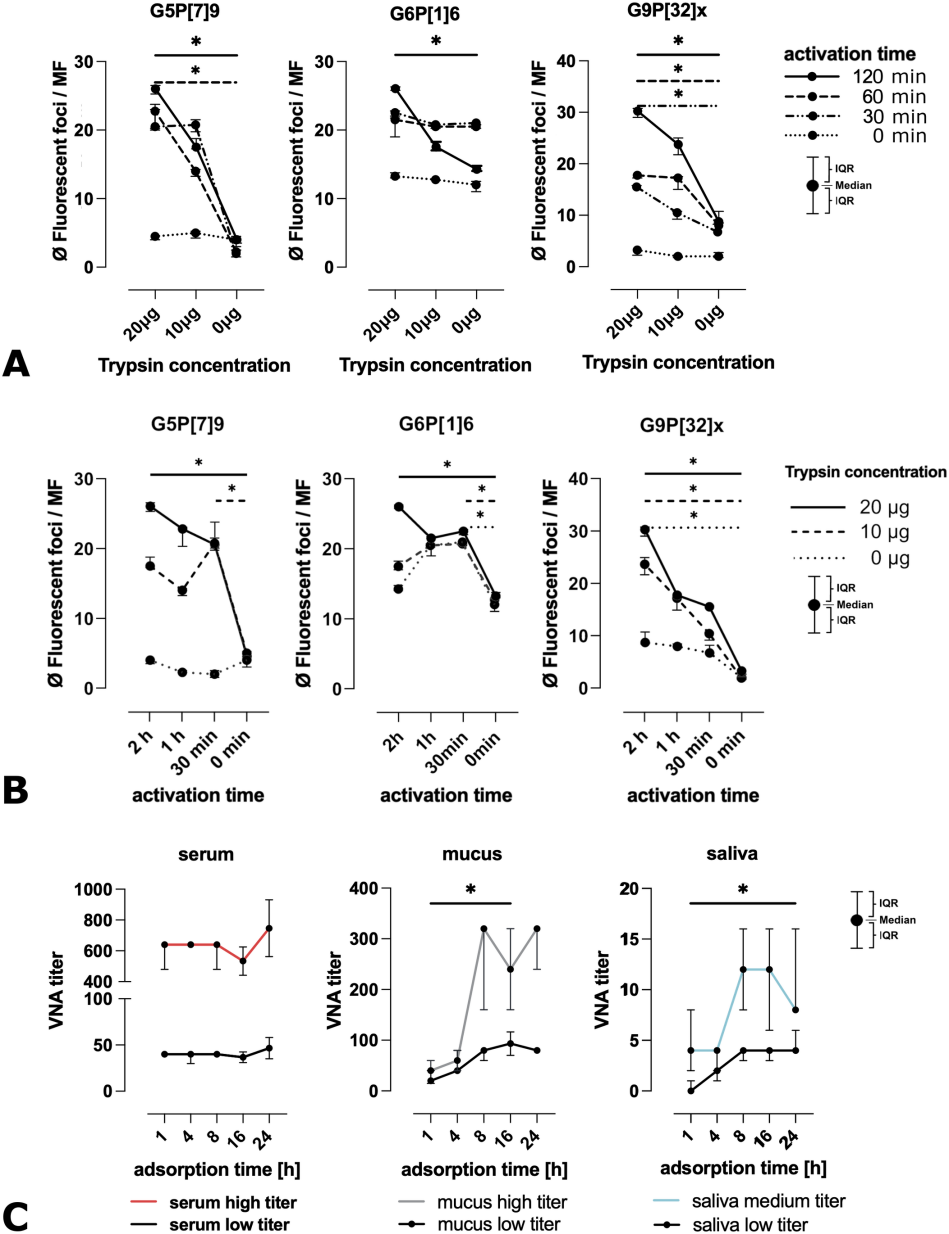
Investigation of virus activation parameters and adsorption time. **(A)** Infectivity of RVA strains depending on trypsin concentration: The figure shows the average number of foci at different trypsin concentrations and an incubation time of 2 h (solid line), 1 h (dashed line), 30 min (dash-dot line) and 0 min (dotted line). For all RVA strains, an increase in the number of foci was observed between the trypsin concentrations from 0 µg/ml to 20 µg/ml. Significant changes were observed for RVA G9P[32]x at incubation times greater than 0 min, for RVA G6P[1]6 at incubation times of 2 h and for RVA G5P[7]9 at incubation times of 2 h and 1 h (legend: MF: microscopic field of view, ⌀: arithmetic mean; representation: median with IQR, Kruskal-Wallis test, n=3). **(B)** Infectivity of RVA strains depending on the incubation time for proteolytic activation: The figure shows the average number of foci at different incubation times for proteolytic activation of RVA at trypsin concentrations of 20 µg/ml (solid line), 10 µg/ml (dashed line) and without trypsin (dotted line). For the RVA G9P[32]x, a significant difference in the number of foci between the incubation time of 0 min and 2 h could be recognized for all trypsin concentrations. This was also observed for the other RVA strains at a trypsin concentration of 20 µg/ml. The RVA G5P[7]9 showed a significant difference between the incubation times of 0 min and 30 min at a trypsin concentration of 10 µg/ml and for RVA G6P[1]6 at a trypsin concentration of 10 µg/ml and without trypsin (legend: MF: microscopic field of view, ⌀: arithmetic mean; representation: median with IQR, Kruskal-Wallis test, n=3). **(C)** VNA titers as a function of the virus-antibody adsorption time: The figures show the VNA titers for two serum samples (left), two saliva samples (center) and two intestinal mucus samples (right) as a function of the adsorption time between the virus and the antibodies contained in sample materials. One sample with a low (black) and one with a higher (colored) VNA titer were used. No significant differences were found in the analysis of the serum samples. The saliva samples and intestinal mucus samples showed higher VNA titers from a time point of 8 h with some significant differences in mucus and saliva samples with a low VNA titer (representation of the median with IQR, Kruskal-Wallis test, n=3; significance level: *p < 0.05).

The duration of proteolytic activation and the composition of the culture medium influenced the average number of fluorescent foci observed in direct immunofluorescence assays (IFAs). The number of foci increased with higher trypsin concentrations (**Figure 2A**). A statistically significant increase in foci was observed for all RVA at a trypsin concentration of 20 µg/ml compared to medium without trypsin. While this effect can be observed for RVA G9P[32]x for all proteolytic activation times (p_120min_ = 0.0219, p_60min_ = 0.0382, p_30min_ = 0.0136) for G6P[1]6 it is shown at 120 minutes (p = 0.0219), and for G5P[7]9 at both 60 and 120 minutes activation time (p = 0.0211 for both). For G9P[32]x, a significant increase was also detected at 60 minutes incubation (p = 0.0382) and 30 minutes incubation (p = 0.0136).

Across all RVA strains, the number of fluorescent foci increased significantly following a two hours proteolytic activation time with 20 µg/ml trypsin (G9P[32]x: p = 0.0127; G6P[1]6: p = 0.0134; G5P[7]9: p = 0.0132) (**Figure 2B**). For RVA G9P[32]x, this increase was also significant at all other trypsin concentrations tested (10 µg/ml: p = 0.0132; 0 µg/ml: p = 0.0153). The number of fluorescent foci for strains G6P[1]6 and G5P[7]9 decreased with prolonged proteolytic activation time after the initial significant increase from 0–30 min at trypsin concentrations of 10 µg/ml (G6P[1]6: p = 0.0289, G5P[7]9: p = 0.0134). A similar trend was observed for G6P[1]6 in absence of trypsin (p = 0.0156). Across all RVA strains, the lowest number of fluorescent foci was observed without any activation time, regardless of the trypsin concentration applied.

In the VNA, no significant changes in neutralization titers over time were observed for serum samples. However, for saliva and intestinal mucus samples, VNA titers increased between 1 and 8 hours of virus–sample adsorption. After 24 hours, titers remained comparable to those at 8 hours. Only in samples with initially low VNA titers, statistically significant differences were observed for saliva between 1 and 24 hours (p = 0.0393), and for intestinal mucus between 1 and 16 hours (p = 0.0287) (**Figure 2C**). Occasionally, mucus samples exhibited bacterial or fungal overgrowth after 16 or 24 hours of adsorption. Furthermore, no statistically significant differences (Kruskal-Wallis test) were detected at the 8-, 16-, or 24-hour time points. Based on these findings, an adsorption time of two hours for serum, and eight hours for saliva and intestinal mucus, was established as optimal in the VNA protocol.

The determination of the minimum multiplicity of infection (MOI) for RVA strain G5P[7]9 using a negative control serum (NC). At MOIs below 3 × 10^-4^, no green fluorescent signals could be detected in the cytoplasm of cells, even at low dilution levels of the negative control serum. At MOI of 3 x 10^-3^, fluorescent foci were still detectable at 10-fold dilution of negative samples (**Supplementary Material 2**). For all virus strains used, the virus-positive control was adjusted to yield 10–20 foci per microscopic field (MF). Based on these settings, the MOIs were calculated assuming the seeded cell number of 0.4 × 10^5^ cells/well and are listed in **Table 3**.

**Table 3:** MOI used in VNA.

RV-group with G- und P-genotype/serotype	Virus concentration [MOI]
RVA G9P[32]x	0.50 x 10^-3^
RVA G6P[1]6	1.25 x 10^-3^
RVA G5P[7]9	3.00 x 10^-3^
RVC G1P[1]1	3.00 x 10^-3^

MOI, Multiplicity of Infection; RVA, Rotavirus group A; RVC, Rotavirus group C.

**Figure 3:**
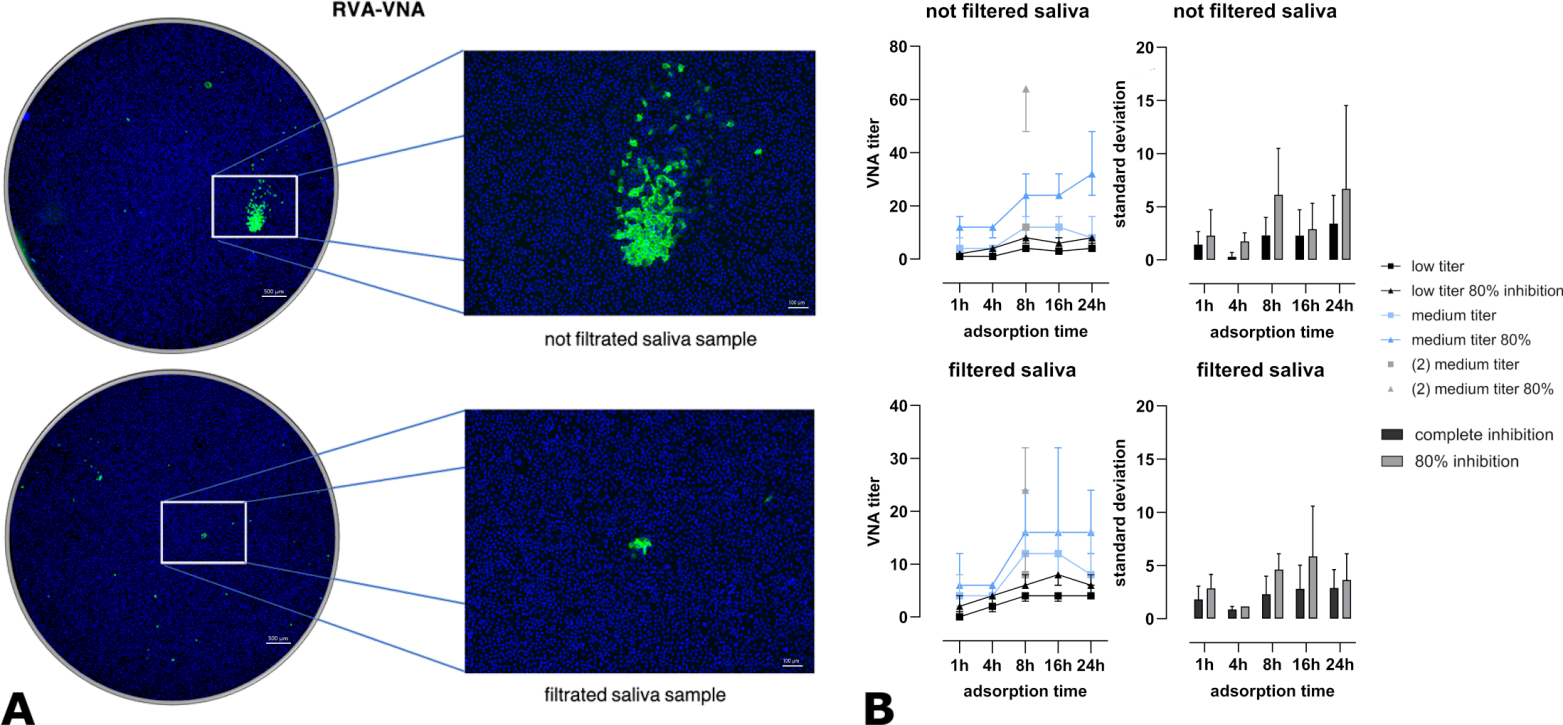
Influence of inhibition rate on read out results. **(A)** Appearance of the fluorescence signals in the saliva-specific VNA: The images on the left show two wells of an RVA-VNA of a 1:4 diluted saliva sample. The cell nuclei (blue) and the specific coloring of the VP6 protein of the RVA (green) can be seen. The upper images show the result of the unfiltered saliva sample. Fluorescent cells are concentrated focally. The lower image shows the result of the same saliva sample after filtration. Only at one point appear a few focally concentrated fluorescent cells. Overall, more individual fluorescent cells appear in this cavity. **(B)** Left: VNA titers of one saliva sample with a low titer and two with medium titers (median ± IQR). A significant difference between readout methods was found for the medium-titer saliva sample 2 at 8 h of adsorption (*t*-test, *p* < 0.005). Right: deviation among triplicates. The 80% readout showed significantly higher variance than the 100% readout (paired *t*-test, *p* = 0.0476).

Besides this sample-specific factors affected assay performance. The appearance of immunofluorescence signals differed depending on the type of sample material. When serum samples were tested in the virus neutralization assay (VNA), green fluorescent signals were clearly associated with individual cells. In contrast, saliva and intestinal mucus samples often produced localized foci comprising clusters of adjacent fluorescent cells, which were counted as a single focus. This effect was markedly reduced after filtration of the saliva samples through a 0.45 µm sterile filter, resulting in more dispersed and individually fluorescent cells (**Figure 3A**). Despite the morphological differences, both filtered and unfiltered saliva samples showed almost identical VNA titers with complete (100%) neutralization. Using an 80% inhibition rate as the readout increased the calculated neutralization titers; however, it was associated with a significantly higher standard deviation among triplicates compared with the 100% inhibition readout (**Figure 3B**).

**Figure 4:**
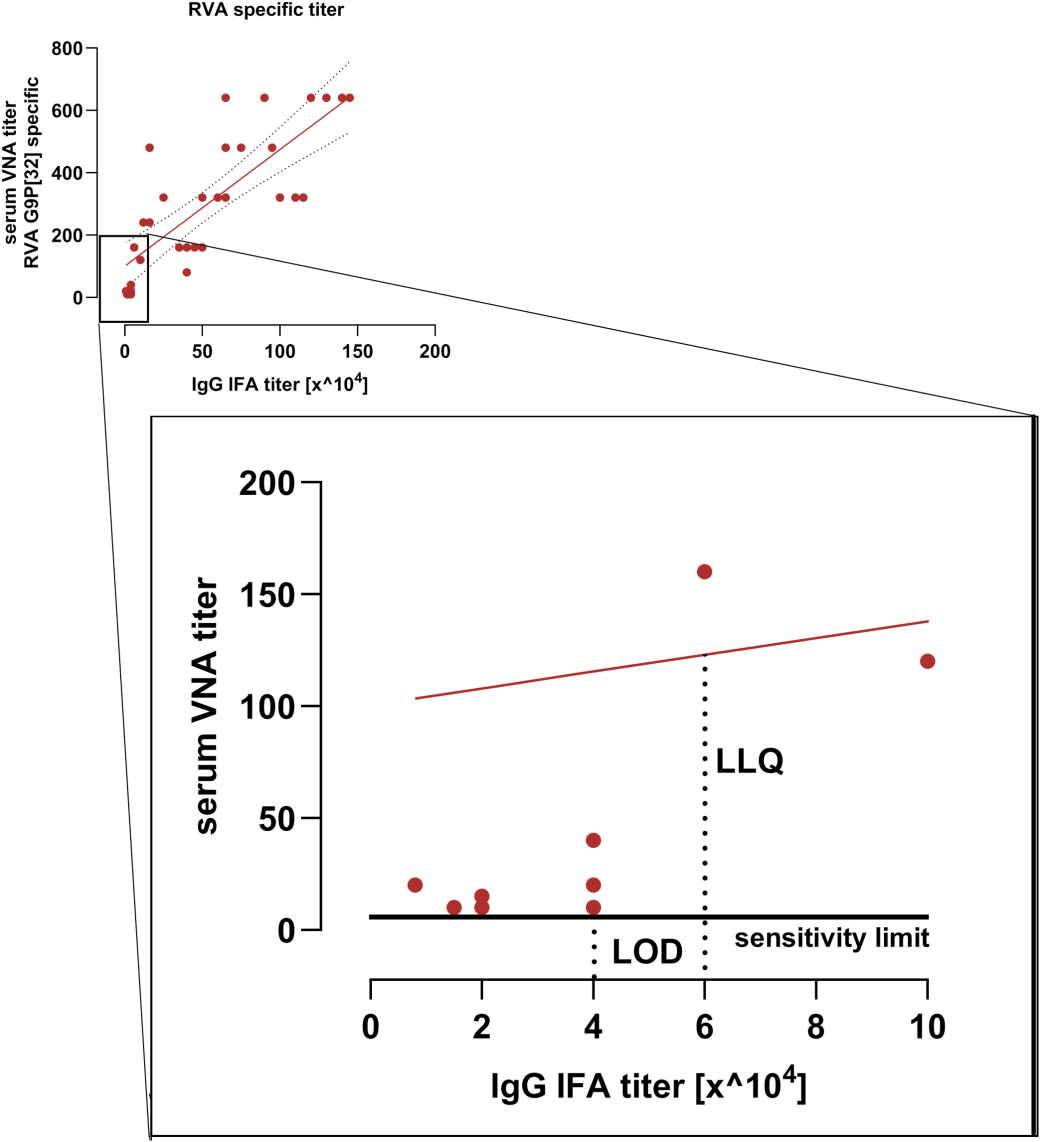
Sensitivity limit and linear range of the serum VNA. Shown is the correlation between RVA-specific IgG IFA titers and VNA titers from the immunization study. The low-titer range is enlarged. The limit of detection (LOD) corresponds to a RVA-specific IgG IFA titer of 40,000. The lower limit of quantification (LLQ) corresponds to a RVA-specific IgG IFA titer of 60.000. The red line indicates the VNA regression line.

The limit of detection (LOD) for the serum VNA corresponds to RVA-specific IgG IFA titer of 4 x 10^4^. The lower limit of quantification (LLQ) is defined at RVA-specific IgG IFA titer of 6 x 10^4^ (**Figure 4**). It has, however, to be taken into account that IFA detects all antigen specific IgG antibodies whereas the VNA detects serotype specific antibodies of all immunoglobulin isotypes. Therefore LOD and LOQ can only be determined approximately.

The inter-assay variation of the VNA showed coefficients of variation (CV) below 15% for sera with medium and high VNA titers. For sera with low titers, the inter-assay CV was 23.4%. In intra-assay comparisons, the coefficients of variation for all samples remained below 10% (**Table 4**).

**Table 4:** Repeatability parameters of the RVA G5P[7]9-specific VNA for serum.

Characteristics sample material		Inter-test	Intra-test
Serum VNA-titer	$\bar{\text{X}}$	V (%)	V (%)
Low titer	2.059	23.4	3.64
median titer	4.858	10.4	1.81
high titer	6.038	6.2	7.58

Information on the inter- and intra-test parameters, indicating the coefficient of variation (V) and the mean value ( $\bar{\text{X}}$) of the normalized titers are shown. The values are presented for one serum each with low, medium and high VNA titers. The ratios were determined after linearization of VNA titers.

$\bar{\text{X}}$, mean value; V, coefficient of variation.

The ratios for saliva samples were determined using low VNA titers, while those for intestinal mucus were assessed using both low and high VNA titers. Coefficients of variation comparable to those observed for serum samples were found (**Table 5**).

**Table 5:** Repeatability parameters of the RVA G5P[7]9-specific VNA for saliva and gut mucus.

Characteristics sample material		Inter-test	Intra-test
sample material	$\bar{\text{X}}$	V (%)	V (%)
saliva	1.590	24.3	4.97
intestinal mucus	2.358	18.2	3.19
intestinal mucus	7.438	4.9	4.25

Information on the inter- and intra-test parameters, indicating the coefficient of variation (V) and the mean value ( $\bar{\text{X}}$) of the normalized titers are shown. The values are presented for a saliva sample with a low VNA titer and an intestinal mucus sample with a low and high VNA titer.

Within the protocol, there are key factors that, if not taken into account, can influence the results of the assay. They are mentioned in **Table 6**.

**Table 6:** Trouble shooting.

Trouble shooting	Description solution
cell layer is not confluent	control trypsin concentration in the activation medium and ensure enough activation time
cells have a spindle-shaped appearance	Trypsin concentration is higher than recommended
no or low infectivity of RVA or RVC in positive control	Remaining FBS in the media have neutralization effect - check the washing procedure before inoculation Wrong MOI used
Titers of saliva or mucus samples are lower than expected	control virus neutralization time check for sample transport at 4 °C and long term storage at -80 °C

### 4.2 Evaluation of an indirect correlate to the gut mucus

Four and six pigs showed RVA G5P[7]9-specific VNA titers below 10 in serum and saliva, respectively, and were therefore excluded from correlation analysis. Salivary RVA G5P[7]9-specific VNA titers correlated strongly with intestinal mucus titers from the duodenum, jejunum, and ileum, with correlation coefficients of 0.96, 0.88, and 0.98, respectively (p = 0.0004, 0.008, and 0.001). In contrast, serum VNA titers showed lower correlations with intestinal mucus titers from the same regions, with coefficients of 0.72, 0.67, and 0.84 (p = 0.0224, 0.041, and 0.004, respectively). A summary of these RVA-specific correlation analyses is provided in **Supplementary Material 3**.

**Figure 5:**
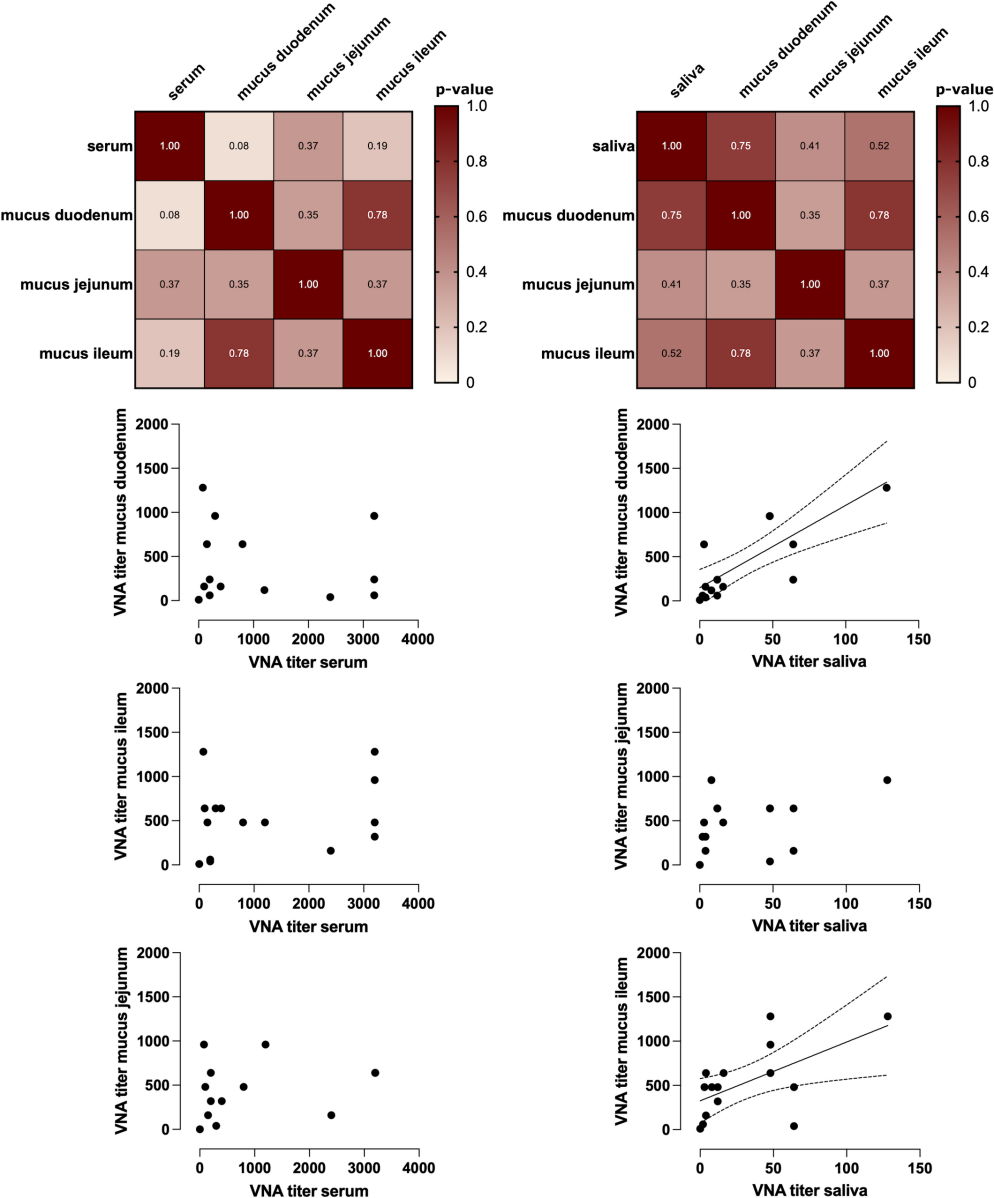
Correlation calculations between RVC-specific VNA titers within sample materials. The correlations between the intestinal mucus VNA titers and the saliva VNA titers or the serum VNA titers are shown graphically in the form of XY plots and a heat map. A correlation line is shown for correlations ≥ 0.5 (Spearman correlation, n = 15).

The strongest correlation of RVC G1P[1]1-specific VNA titers was observed between saliva and duodenal intestinal mucus from the same animals, with a correlation coefficient of 0.75 (p = 0.0058). Further analysis revealed lower, non-significant correlations between salivary VNA titers and intestinal mucus from the ileum (r= 0.50) and jejunum (r = 0.42; p > 0.05 for both). Conversely, serum VNA titers showed the highest correlation with jejunal intestinal mucus (r = 0.49), followed by ileal (r = 0.32) and duodenal mucus (r= 0.09), none of which reached statistical significance (p > 0.05). Overall, correlations between salivary VNA titers and intestinal mucus were higher and more significant than those observed for serum. The RVC-specific correlation results are summarized in **Figure 5**.

### 4.3 Evaluation of VNA and IFA titers in the vaccination study

Samples from vaccinated pigs were analyzed to determine the correlation between serum RVA-specific IgA IFA titers and salivary neutralizing titers. Additionally, the relationship between serum RVA-specific IgG IFA titers and RVA G9P[32]x-specific serum neutralizing titers was evaluated.

**Figure 6:**
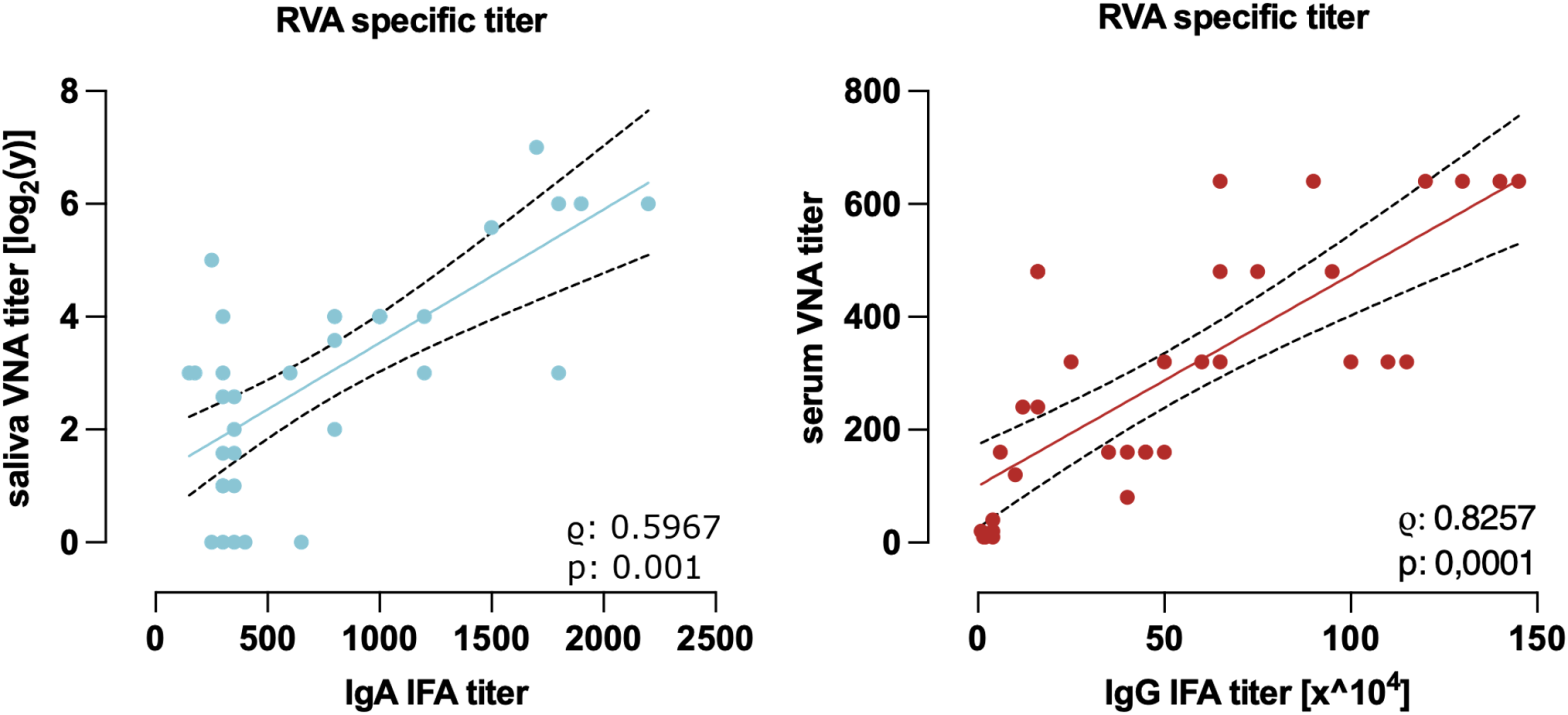
Correlation of the RVA IgG and IgA IFA titers with the VNA titers in serum and saliva. The correlation between the RVA-specific IgG IFA titers and the G9P[32]x specific VNA titers in serum (left) and the RVA-specific IgA IFA titers in serum and the G9P[32]x specific VNA titers in saliva (right) is shown. The correlations are strongly positive with a value of 0.8257 (Spearman correlation) coefficient and 0.7098 (Pearson correlation) with a significance of < 0.0001 (representation of the regression line with 95% confidence interval in casas of correlation > 0.5, n = 35).

A strong correlation was observed between serum RVA-specific IgG IFA titers and G9P[32]x-specific serum VNA titers (r = 0.8257, p < 0.0001). Likewise, serum IgA IFA titers correlated positively with log_2_-transformed salivary G9P[32]x-specific VNA titers (Pearson correlation, r = 0.7098, p < 0.0001), as shown in **Figure 6**.

## 5 Discussion

There is currently no universally accepted VNA protocol, as each assay is influenced by pathogen-specific characteristics and the properties of the sample material (26–29). Consequently, each new protocol must be validated based on defined parameters and subjected to an optimization process.

In the present study, five factors were identified that significantly influence the outcome of a RV-specific VNA: (i) trypsin concentration, (ii) incubation time for proteolytic activation, (iii) infection dose, (iv) neutralization time, and (v) sample material-specific factors.

The RVA strains used exhibited strain-specific, concentration- and time-dependent changes in infectivity upon proteolytic activation with trypsin. This observation is consistent with findings from earlier studies (33–36). As an endopeptidase, trypsin cleaves peptide bonds at basic amino acid residues. Two conserved cleavage sites within the VP4 protein of RVA facilitate trypsin-mediated conformational changes critical for viral entry (37, 38). Despite these conserved cleavage motifs, the reasons for the observed variability in trypsin sensitivity among RVA strains remain unclear. Mammalian cells typically respond to viral infection *in vitro* by secreting interferon, a key antiviral cytokine. The RVA non-structural protein NSP1 functions as an interferon antagonist (39), and its 39 known genotypes exhibit variable efficacy in suppressing interferon responses (40). Trypsin has also been shown to directly inhibit interferon activity (41). For strains with NSP1 variants that weakly antagonize interferon signaling, trypsin-mediated inactivation of interferon may enhance infectivity (41). This hypothesis should be tested by incorporating interferon inhibitors into the assay.

VNA titers can be interpreted based on complete or partial inhibition of infection. Even among virus-specific protocols from different laboratories, inconsistent readout methods are observed (42, 43), including for RVA-specific VNA (44–46). The choice of readout threshold can affect test sensitivity and specificity (29). Given the need for high sensitivity in the analysis of saliva samples, partial inhibition was initially selected as the preferred readout method. However, viral infection patterns changed when using saliva or intestinal mucus compared to serum. While single infected cells were observable in serum and sterile-filtered saliva, unfiltered saliva and intestinal mucus yielded focally clustered infections. Moreover, VNA titers determined by the 80% readout method exhibited significantly greater variability among triplicates. Therefore, we chose to interpret VNA titers based on complete inhibition of infection.

Mucins in saliva and intestinal mucus have high affinity for positively charged ions such as calcium (47). These large, complex glycoproteins, fragmented and homogenized via bead mill treatment, may distribute unevenly in the sample, resulting in focal accumulation. Calcium ions, in turn, stabilize the outer capsid layer of RV (48), potentially facilitating localized infection of multiple cells and contributing to focus formation. On the other hand, certain mucins have demonstrated RV-inhibitory activity (49–52). Mucin-covered areas may be resistant to infection, concentrating infection in mucin-free regions. Further investigation is required to prove this hypothesis and to determine which mechanism may predominate. The disappearance of foci following sterile filtration of saliva and removal of high-molecular-weight mucins (53) supports the hypothesis of mucin involvement. To further support the hypothesis, analyses involving mucin digestion methods, calcium staining protocols or electron microscopic investigation should be conducted. Despite the sensitivity loss associated with the selected readout method, specific antibodies were detectable in saliva. A critical factor enabling this was the adjustment of the neutralization time. Since many other studies did not adjust the neutralization time (54, 55), potentially influenced their results, this approach may yield different outcomes. While neutralization time had minimal effect on serum titers, it proved decisive for saliva and intestinal mucus samples. Secretory immunoglobulins (SIgs) exhibit higher avidity for antigens than monomeric serum immunoglobulins (56, 57). Nonetheless, SIgs do not diffuse freely in mucosal environments; rather, they form reversible associations with mucins (58, 59). This limits their mobility, delaying antigen encounter and potentially sequestering the virus within the mucus matrix, thus preventing cellular entry (52). This mechanism may also involve non-neutralizing antibodies that block infection through steric hindrance or aggregation (60, 61). Consequently, VNAs using saliva or intestinal mucus can detect both neutralizing and functionally relevant non-neutralizing antibodies (62, 63).

All these factors influence the maximum RV-specific focus formation in the VNA and therefore affect the neutralization titers measured. This highlights the critical need to consider both pathogen- and sample-specific variables to ensure reliable assay performance.

Repeatability ratios met OIE standards (OIE, 2009), with inter-assay coefficients of variation below 15% for sera with moderate to high titers and intra-assay variation below 10%. However, in low titer ranges, variation exceeded OIE recommendations. Correlation analysis revealed that RVA-specific serum VNA titers deviated from linearity at low concentrations, indicating the assay’s sensitivity threshold had been reached. While high and consistent titers were obtained from serum and intestinal mucus, saliva samples yielded significantly lower and more variable titers. This is attributable to the substantially lower relative concentrations of IgG and SIgA in saliva—20-fold and 3000-fold lower, respectively, than in serum (15, 64, 65), and approximately 1000-fold lower than in intestinal mucus (66)—resulting in an inherent loss of sensitivity for this matrix.

Nonetheless, the RV-specific titers detected in saliva align with data from validation studies for Porcine Reproductive and Respiratory Syndrome virus (67) and Schmallenberg virus (26). Nevertheless, in situations where immune responses are weak or suboptimal, reliance on whole saliva based VNA measurements alone may not provide a comprehensive evaluation of the immune status. In pigs, TGEV-specific B cells were detected in higher numbers in the submandibular and sublingual salivary glands compared to the parotid gland (68). In humans, a correlation between IgA concentrations in *submandibular* and *sublingual* glands and the enteric mucosal immune status has been demonstrated (69). Therefore, normalization of IgA concentrations and neutralizing antibody titers obtained from saliva samples to the respective proportions of the *submandibular* and *sublingual* salivary gland secretions would be a desirable approach to refine the assessment of the immunological status. The combined assessment of RV-specific IgA concentrations in serum or saliva and virus neutralization activity (VNA) may enhance the evaluation of the immune status and thereby addresses the inherent limitation of the VNA in its inability to discriminate between different immunoglobulin isotypes. In humans, standardized protocols for sampling this specific salivary fraction have been established (69), suggesting that the application of VNA may be more appropriate in humans than in pigs. Thus, defining a salivary reference for neutralization titer normalization in pigs should be further developed for broader clinical application.

The protocol established in this study currently represents the most optimized method for detecting RV-specific neutralizing antibodies in the tested matrices. However, standardization of diagnostic protocols across laboratories is essential to ensure comparability between studies. Moreover, this assay does not address cellular immune responses—an essential component of immunity against rotavirus (70–72), highlighting the need for a multimodal diagnostic approach.

In pigs, evidence supports the existence of mucosal immune system cross-talk (73, 74). The data presented here further support this, with findings indicating stronger correlations between salivary and intestinal immune responses in more proximal intestinal segments, consistent with human studies (75).

In contrast, serum-derived RV-specific neutralizing antibody titers do not reliably reflect the immune status of the intestinal mucosa. VNAs do not distinguish between immunoglobulin classes and therefore predominantly reflect the contribution of IgG, the major serum antibody, which may mask the effects of IgA and IgM. However, correlations between mucosal immunity and RV-specific IgA levels in blood have been demonstrated in pigs (72), humans (76), and mice (77). In this study, a significant correlation between serum IgA concentrations and salivary titers was also observed, reinforcing these previous findings.

Despite their compositional variability, saliva samples offer a minimally invasive and practical diagnostic tool (19, 21–23). The validated VNA protocol presented here demonstrates the utility of saliva in monitoring immune responses and highlights its potential for broader application, provided a robust and standardized assay is available. This approach would also facilitate longitudinal immune monitoring and reduce the number of animals required in vaccine studies, thereby aligning with ethical principles of animal use reduction.

## Data Availability

The datasets presented in this study can be found in online repositories. The names of the repository/repositories and accession number(s) can be found below: DOI: https://doi.org/10.25532/OPARA-906.

## Footnotes

1 For Original Research articles, please note that the Material and Methods section can be placed in any of the following ways: before Results, before Discussion or after Discussion.

## Supplementary material

Supplementary Material 1 Detailed final protocol.

Supplementary Material 2 Determination of the Optimal Multiplicity of Infection (MOI). Virus loads corresponding to MOIs of 3 × 10^-2^ (left), 3 × 10^-3^ (center), and 3 × 10^-4^ (right) were used in the virus neutralization assay (VNA). As the serum negative controll was progressively diluted, the number of foci per well increased accordingly. At MOIs of 3 × 10^-2^ and 3 × 10^-3^, serum negative controll showed a VNA titer < 10. In contrast, at an MOI of 3 × 10^-4^, the VNA titer increased to 40.

Supplementary Material 3 Correlation calculations between RVA-specific VNA titers within sample materials. The correlations between the intestinal mucus VNA titers and the saliva VNA titers or the serum VNA titers are shown graphically in the form of XY plots and a heat map. A correlation line is shown for correlations ≥ 0.5 (Spearman correlation, n = 5 or 7).

## References

[1] Bladh O Aguilera K Marking U Kihlgren M Greilert Norin N Smed-Sörensen A . Comparison of SARS-CoV-2 spike-specific IgA and IgG in nasal secretions, saliva and serum. Front Immunol. (2024) 15:1346749. doi: 10.3389/fimmu.2024.1346749, PMID: 38558811 PMC10978617

[2] Guerra ENS Castro VTD dos Santos JA Acevedo AC Chardin H . Saliva is suitable for SARS-CoV-2 antibodies detection after vaccination: a rapid systematic review. Front Immunol. (2022) 13:1006040. doi: 10.3389/fimmu.2022.1006040, PMID: 36203571 PMC9530471

[3] Koch T Mellinghoff SCM Shamsrizi P Addo MM Dahlke C . Correlates of vaccine-induced protection against SARS-CoV-2. Vaccines. (2021) 9:238. doi: 10.3390/vaccines9030238, PMID: 33801831 PMC8035658

[4] Lamm ME . Interaction of antigens and antibodies at mucosal surfaces. Annu Rev Microbiol. (1997) 51:311–40. doi: 10.1146/annurev.micro.51.1.311, PMID: 9343353

[5] Brandtzaeg P . Gate-keeper function of the intestinal epithelium. Benef Microbes. (2013) 4:67–82. doi: 10.3920/BM2012.0024, PMID: 23257015

[6] Neutra MR Kozlowski PA . Mucosal vaccines: the promise and the challenge. Nat Rev Immunol. (2006) 6:148–58. doi: 10.1038/nri1777, PMID: 16491139

[7] Hjelt K Grauballe C . Protective levels of intestinal rotavirus antibodies. J Infect Dis. (1990) 161:352–53. doi: 10.1093/infdis/161.2.352, PMID: 2153741

[8] Coulson BS Grimwood K Hudson IL Barnes GL Bishop RF . Role of coproantibody in clinical protection of children during reinfection with rotavirus. J Clin Microbiol. (1992) 30:1678–84. doi: 10.1128/jcm.30.7.1678-1684.1992, PMID: 1321167 PMC265363

[9] O'Neal CM Crawford SE Estes MK Conner ME . Rotavirus virus-like particles administered mucosally induce protective immunity. J Virol. (1997) 71:8707–17. doi: 10.1128/JVI.71.11.8707-8717.1997, PMID: 9343229 PMC192335

[10] Parez N Fourgeux C Mohamed A Dubuquoy C Pillot M Dehee A . Rectal immunization with rotavirus virus-like particles induces systemic and mucosal humoral immune responses and protects mice against rotavirus infection. J Virol. (2006) 80:1752–61. doi: 10.1128/JVI.80.4.1752-1761.2006, PMID: 16439532 PMC1367137

[11] Plotkin SA Gilbert PB . Nomenclature for immune correlates of protection after vaccination. Clin Infect Dis. (2012) 54:1615–17. doi: 10.1093/cid/cis238, PMID: 22437237 PMC3348952

[12] Brandtzaeg P . Mucosal immunity: induction, dissemination, and effector functions. Scand J Immunol. (2009) 70:505–15. doi: 10.1111/j.1365-3083.2009.02319.x, PMID: 19906191

[13] Samaranayake LL . Saliva as a diagnostic fluid. Int Dent J. (2007) 57:295–99. doi: 10.1111/j.1875-595X.2007.tb00135.x, PMID: 17992912

[14] Prickett JR Zimmerman JJ . The development of oral fluid-based diagnostics and applications in veterinary medicine. Anim Health Res Rev. (2010) 11:207–16. doi: 10.1017/S1466252310000010, PMID: 20202287

[15] Decorte I van Breedam W van der Stede Y Nauwynck HJ de Regge N Cay AB . Detection of total and PRRSV-specific antibodies in oral fluids collected with different rope types from PRRSV-vaccinated and experimentally infected pigs. BMC Vet Res. (2014) 10:134. doi: 10.1186/1746-6148-10-134, PMID: 24938323 PMC4072892

[16] Grau FR Schroeder ME Mulhern EL McIntosh MT Bounpheng MA . Detection of African swine fever, classical swine fever, and foot-and-mouth disease viruses in swine oral fluids by multiplex reverse transcription real-time PCR. J Vet Diagn Invest. (2015) 27:140–49. doi: 10.1177/1040638715574768, PMID: 25776540

[17] Senthilkumaran C Yang M Bittner H Ambagala A Lung O Zimmerman JJ . Detection of genome, antigen, and antibodies in oral fluids from pigs infected with foot-and-mouth disease virus. Can J Vet Res. (2017) 81:82–90., PMID: 28408775 PMC5370543

[18] Atkinson JC . Guidelines for saliva nomenclature and collection. Ann N Y Acad Sci. (1993) 694:xi–xii. doi: 10.1111/j.1749-6632.1993.tb18335.x

[19] Olsen C Karriker L Wang C Binjawadagi B Renukaradhya GK Kittawornrat A . Effect of collection material and sample processing on pig oral fluid testing results. Vet J. (2013) 198:158–63. doi: 10.1016/j.tvjl.2013.06.014, PMID: 24011474

[20] Booth CK Dwyer DB Pacque PF Ball MJ . Measurement of immunoglobulin A in saliva by particle enhanced nephelometric immunoassay: sample collection, limits of quantitation, precision, stability and reference range. Ann Clin Biochem. (2009) 46:401–06. doi: 10.1258/acb.2009.008248, PMID: 19641004

[21] Rudney JD Smith QT . Relationships between levels of lysozyme, lactoferrin, salivary peroxidase, and secretory immunoglobulin A in stimulated parotid saliva. Infect Immun. (1985) 49:469–75. doi: 10.1128/IAI.49.3.469-475.1985, PMID: 4030086 PMC261184

[22] Muneta Y Yoshikawa T Minagawa Y Shibahara T Maeda R Omata Y . Salivary IgA as a useful non-invasive marker for restraint stress in pigs. J Vet Med Sci. (2010) 72:1295–300. doi: 10.1292/jvms.10-0009, PMID: 20467204

[23] Brandtzaeg P . Secretory immunity with special reference to the oral cavity. J Oral Microbiol. (2013) 5:20401. doi: 10.3402/jom.v5i0.20401, PMID: 23487566 PMC3595421

[24] Page LJ Lagunas Acosta J Castellana ET Messmer BT . Accurate prediction of serum antibody levels from noninvasive saliva/nasal samples. BioTechniques. (2023) 74:131–36. doi: 10.2144/btn-2022-0106, PMID: 37038960

[25] Angel J Steele AD Franco MA . Correlates of protection for rotavirus vaccines: possible alternative trial endpoints, opportunities, and challenges. Hum Vaccin Immunother. (2014) 10:3659–67. doi: 10.4161/21645515.2014.972172, PMID: 25483685 PMC4514048

[26] Loeffen W Quak S de Boer Luijtze E Hulst M van der Poel W Bouwstra RB . Development of a virus neutralisation test to detect antibodies against Schmallenberg virus and serological results in suspect and infected herds. Acta Vet Scand. (2012) 54:44. doi: 10.1186/1751-0147-54-44, PMID: 22871162 PMC3503834

[27] Vaidya SR Brown DW Jin L Samuel D Andrews N Brown KE . Development of a focus reduction neutralization test (FRNT) for detection of mumps virus neutralizing antibodies. J Virol Methods. (2010) 163:153–56. doi: 10.1016/j.jviromet.2009.09.006, PMID: 19761798

[28] Bedeković T Lemo N Lojkić I Mihaljević Ž Jungić A Cvetnić Ž . Modification of the fluorescent antibody virus neutralisation test—elimination of the cytotoxic effect for the detection of rabies virus neutralising antibodies. J Virol Methods. (2013) 189:204–08. doi: 10.1016/j.jviromet.2013.01.022, PMID: 23403247

[29] Meyer B Reimerink J Torriani G Brouwer F Godeke GJ Yerly S . Validation and clinical evaluation of a SARS-CoV-2 surrogate virus neutralisation test (sVNT). Emerg Microbes Infect. (2020) 9:2394–403. doi: 10.1080/22221751.2020.1835448, PMID: 33043818 PMC7605318

[30] Lee B . Update on rotavirus vaccine underperformance in low-to middle-income countries and next-generation vaccines. Hum Vaccin Immunother. (2021) 17:1787–802. doi: 10.1080/21645515.2020.1844525, PMID: 33327868 PMC8115752

[31] Velasquez DE Parashar U Jiang B . Decreased performance of live attenuated, oral rotavirus vaccines in low-income settings: causes and contributing factors. Expert Rev Vaccines. (2018) 17:145–61. doi: 10.1080/14760584.2018.1409394, PMID: 29252042 PMC8826512

[32] StIKoVet Stellungnahme bestandsspezifischer-Impfstoffe. (PDF) . OpenAgrar. Available online at: https://www.openagrar.de/servlets/MCRFileNodeServlet/openagrar_derivate_00028622/StIKoVet_Stellungnahme_bestandsspezifischer-Impfstoffe.pdf (Accessed November 1, 2023).

[33] Arias CF Romero P Alvarez V López S . Trypsin activation pathway of rotavirus infectivity. J Virol. (1996) 70:5832–39. doi: 10.1128/jvi.70.9.5832-5839.1996, PMID: 8709201 PMC190599

[34] Espejo RT López S Arias CA . Structural polypeptides of simian rotavirus SA11 and the effect of trypsin. J Virol. (1981) 37:156–60. doi: 10.1128/JVI.37.1.156-160.1981, PMID: 6260970 PMC170992

[35] Estes MK Graham DY Mason BB . Proteolytic enhancement of rotavirus infectivity: molecular mechanisms. J Virol. (1981) 39:879–88. doi: 10.1128/JVI.39.3.879-888.1981, PMID: 6270356 PMC171321

[36] Rodríguez JM Chichón FJ Martín Forero E González Camacho F Carrascosa JL Castón JR . New insights into rotavirus entry machinery: stabilization of rotavirus spike conformation is independent of trypsin cleavage. PloS Pathog. (2014) 10:e1004157. doi: 10.1371/journal.ppat.1004157, PMID: 24873828 PMC4038622

[37] López S Arias CF Méndez E Espejo RT . Conservation in rotaviruses of the protein region containing the two sites associated with trypsin enhancement of infectivity. Virology. (1986) 154:224–27. doi: 10.1016/0042-6822(86)90445-9, PMID: 3019004

[38] Kaljot KT Shaw RD Rubin DH Greenberg HB . Infectious rotavirus enters cells by direct cell membrane penetration, not by endocytosis. J Virol. (1988) 62:1136–44. doi: 10.1128/JVI.62.4.1136-1144.1988, PMID: 2831376 PMC253121

[39] Barro M Patton JT . Rotavirus NSP1 inhibits expression of type I interferon by antagonizing the function of interferon regulatory factors IRF3, IRF5, and IRF7. J Virol. (2007) 81:4473–81. doi: 10.1128/JVI.02498-06, PMID: 17301153 PMC1900170

[40] Arnold MM Sen A Greenberg HB Patton JT . The battle between rotavirus and its host for control of the interferon signaling pathway. PLoS Pathog. (2013) 9:e1003064. doi: 10.1371/journal.ppat.1003064, PMID: 23359266 PMC3554623

[41] Almeida JD Hall T Banatvala JE Totterdell BM Chrystie IL . The effect of trypsin on the growth of rotavirus. J Gen Virol. (1978) 40:213–18. doi: 10.1099/0022-1317-40-1-213, PMID: 211180

[42] Stephenson I Heath A Major D Newman RW Hoschler K Weng J . Reproducibility of serologic assays for influenza virus A (H5N1). Emerg Infect Dis. (2009) 15:1252–59. doi: 10.3201/eid1508.081754, PMID: 19751587 PMC2815968

[43] Thomas SJ Nisalak A Anderson KB Libraty DH Kalayanarooj S Vaughn DW . Dengue plaque reduction neutralization test (PRNT) in primary and secondary dengue virus infections: how alterations in assay conditions impact performance. Am J Trop Med Hyg. (2009) 81:825–33. doi: 10.4269/ajtmh.2009.08-0625, PMID: 19861618 PMC2835862

[44] Coulson BS Grimwood K Masendycz PJ Lund JS Mermelstein N Bishop RF . Comparison of rotavirus immunoglobulin A coproconversion with other indices of rotavirus infection in a longitudinal study in childhood. J Clin Microbiol. (1990) 28:1367–74. doi: 10.1128/jcm.28.6.1367-1374.1990, PMID: 2166082 PMC267934

[45] Jiang B Estes MK Barone C Barniak V O'Neal CM Ottaiano A . Heterotypic protection from rotavirus infection in mice vaccinated with virus like particles. Vaccine. (1999) 17:1005–13. doi: 10.1016/S0264-410X(98)00317-X, PMID: 10067709

[46] Ward RL Kapikian AZ Goldberg KM Knowlton DR Watson WM Rappaport R . Serum rotavirus neutralizing antibody titers compared by plaque reduction and enzyme linked immunosorbent assay based neutralization assays. J Clin Microbiol. (1996) 34:983–85. doi: 10.1128/JCM.34.4.983-985.1996, PMID: 8815124 PMC228933

[47] Kretsinger RH . Calcium binding proteins. Annu Rev Biochem. (1976) 45:239–66. doi: 10.1146/annurev.bi.45.070176.001323, PMID: 134666

[48] Pando V Isa P Arias CF López S . Influence of calcium on the early steps of rotavirus infection. Virology. (2002) 295:190–200. doi: 10.1006/viro.2001.1337, PMID: 12033777

[49] Chen CC Baylor M Bass DM . Murine intestinal mucins inhibit rotavirus infection. Gastroenterology. (1993) 105:84–92. doi: 10.1016/0016-5085(93)90013-3, PMID: 8390382

[50] Willoughby RE Yolken RH . SA11 rotavirus is specifically inhibited by an acetylated sialic acid. J Infect Dis. (1990) 161:116–19. doi: 10.1093/infdis/161.1.116, PMID: 2153181

[51] Yolken RH Ojeh O Khatri IA Sajjan U Forstner JF . Intestinal mucins inhibit rotavirus replication in an oligosaccharide dependent manner. J Infect Dis. (1994) 169:1002–06. doi: 10.1093/infdis/169.5.1002, PMID: 8169384

[52] Raev SA Amimo JO Saif LJ Vlasova AN . Intestinal mucin-type O-glycans: the major players in the host-bacteria-rotavirus interactions. Gut Microbes. (2023) 15:2197833. doi: 10.1080/19490976.2023.2197833, PMID: 37020288 PMC10078158

[53] Bansil R Stanley E LaMont JT . Mucin biophysics. Annu Rev Physiol. (1995) 57:635–57. doi: 10.1146/annurev.ph.57.030195.003223, PMID: 7778881

[54] Kapikian AZ Wyatt RG Levine MM Yolken RH VanKirk DH Dolin R . Oral administration of human rotavirus to volunteers: induction of illness and correlates of resistance. J Infect Dis. (1983) 147:95–106. doi: 10.1093/infdis/147.1.95, PMID: 6296243

[55] Ward RL Bernstein DI Shukla R Young EC Sherwood JR McNeal MM . Effects of antibody to rotavirus on protection of adults challenged with a human rotavirus. J Infect Dis. (1989) 159:79–88. doi: 10.1093/infdis/159.1.79, PMID: 2535868

[56] Johansen FE Braathen R Brandtzaeg P . Role of J chain in secretory immunoglobulin formation. Scand J Immunol. (2000) 52:240–48. doi: 10.1046/j.1365-3083.2000.00790.x, PMID: 10972899

[57] Renegar KB Jackson GD Mestecky J . *In vitro* comparison of the biologic activities of monoclonal monomeric IgA, polymeric IgA, and secretory IgA. J Immunol. (1998) 160:1219–23. doi: 10.4049/jimmunol.160.3.1219, PMID: 9570537

[58] Jensen MA Wang YY Lai SY Forest MG McKinley SA . Antibody mediated immobilization of virions in mucus. Bull Math Biol. (2019) 81:4069–99. doi: 10.1007/s11538-019-00653-6, PMID: 31468263 PMC6764938

[59] Schaefer A Lai SY . The biophysical principles underpinning muco trapping functions of antibodies. Hum Vaccin Immunother. (2022) 18:1939605. doi: 10.1080/21645515.2021.1939605, PMID: 34314289 PMC9116395

[60] Forthal DN . Functions of antibodies. Microbiol Spectr. (2014) 2:10–1128. doi: 10.1128/microbiolspec.AID-0019-2014, PMID: 25215264 PMC4159104

[61] Hope TJ . Moving ahead an HIV vaccine: to neutralize or not, a key HIV vaccine question. Nat Med. (2011) 17:1195–97. doi: 10.1038/nm.2528, PMID: 21988997

[62] Klasse PJ . Neutralization of virus infectivity by antibodies: old problems in new perspectives. Adv Biol. (2014) 2014:157895. doi: 10.1155/2014/157895, PMID: 27099867 PMC4835181

[63] Klasse PJ Sattentau QJ . Occupancy and mechanism in antibody mediated neutralization of animal viruses. J Gen Virol. (2002) 83:2091–108. doi: 10.1099/0022-1317-83-9-2091, PMID: 12185262

[64] Curtis J Bourne FJ . Immunoglobulin quantitation in sow serum, colostrum and milk and the serum of young pigs. Biochim Biophys Acta. (1971) 236:319–32. doi: 10.1016/0005-2795(71)90181-4, PMID: 4102801

[65] Escribano D Gutiérrez AM Subiela Martínez S Tecles F Cerón JJ . Validation of three commercially available immunoassays for quantification of IgA, IgG, and IgM in porcine saliva samples. Res Vet Sci. (2012) 93:682–87. doi: 10.1016/j.rvsc.2011.09.018, PMID: 22019471

[66] Bourne FJ Pickup J Honour JW . Intestinal immunoglobulins in the pig. Biochim Biophys Acta. (1971) 229:18–25. doi: 10.1016/0005-2795(71)90312-6, PMID: 5543606

[67] Ouyang K Binjawadagi B Kittawornrat A Olsen C Hiremath J Elkalifa N . Development and validation of an assay to detect porcine reproductive and respiratory syndrome virus-specific neutralizing antibody titers in pig oral fluid samples. Clin Vaccine Immunol. (2013) 20:1305–13. doi: 10.1128/CVI.00208-13, PMID: 23784856 PMC3754528

[68] De Buysscher EV Berman DT . Secretory immune response in intestinal mucosa and salivary gland after experimental infection of pigs with transmissible gastroenteritis virus. Am J Vet Res. (1980) 41:1214–20. doi: 10.2460/ajvr.41.1214, PMID: 7192522

[69] Aase A Sommerfelt H Petersen LB Bolstad M Cox RJ Langeland N . Salivary IgA from the sublingual compartment as a novel noninvasive proxy for intestinal immune induction. Mucosal Immunol. (2016) 9:884–93. doi: 10.1038/mi.2015.107, PMID: 26509875

[70] Yuan L Saif LJ . Induction of mucosal immune responses and protection against enteric viruses: rotavirus infection of gnotobiotic pigs as a model. Vet Immunol Immunopathol. (2002) 87:147–60. doi: 10.1016/S0165-2427(02)00046-6, PMID: 12072229 PMC7119626

[71] Chepngeno J Amimo JO Michael H Raev SA Jung K Lee MV . Vitamin A deficiency and vitamin A supplementation affect innate and T cell immune responses to rotavirus A infection in a conventional sow model. Front Immunol. (2023) 14:1188757. doi: 10.3389/fimmu.2023.1188757, PMID: 37180172 PMC10166828

[72] Yuan L Ward LA Rosen BI Nguyen ToTL Saif LJ . Systemic and intestinal antibody secreting cell responses and correlates of protective immunity to human rotavirus in a gnotobiotic pig model of disease. J Virol. (1996) 70:3075–83. doi: 10.1128/JVI.70.5.3075-3083.1996, PMID: 8627786 PMC190169

[73] De Buysscher EV Dubois PR . Detection of IgA anti-Escherichia coli plasma cells in the intestine and salivary glands of pigs orally and locally infected with E. coli. In: McGhee JR Mestecky J Babb JL , editors. Secretory Immunity and Infection. Boston, MA: Springer US: AEMB (1978). p. 593–600., PMID: 10.1007/978-1-4684-3369-2_67 369316

[74] Foss DL Murtaugh MP . Mucosal immunogenicity and adjuvanticity of cholera toxin in swine. Vaccine. (1999) 17:788–801. doi: 10.1016/S0264-410X(98)00263-1, PMID: 10067684

[75] Brandtzaeg P . Do salivary antibodies reliably reflect both mucosal and systemic immunity? Ann N Y Acad Sci. (2007) 1098:288–311. doi: 10.1196/annals.1384.012, PMID: 17435136

[76] Ward RL Knowlton DR Zito ET Davidson BL Rappaport R Mack ME . Serologic correlates of immunity in a tetravalent reassortant rotavirus vaccine trial. J Infect Dis. (1997) 176:570–77. doi: 10.1086/514076, PMID: 9291301

[77] McNeal MM Broome RL Ward RL . Active immunity against rotavirus infection in mice is correlated with viral replication and titers of serum rotavirus IgA following vaccination. Virology. (1994) 204:642–50. doi: 10.1006/viro.1994.1579, PMID: 7941332

