# Supplementary figures and images for: Rotavirus-specific neutralization assay enables evaluation of mucosal immune responses
Source: Front Immunol. 2025 Dec 2;16:1677823. doi: 10.3389/fimmu.2025.1677823 (PMC12706807; doi:10.3389/fimmu.2025.1677823)

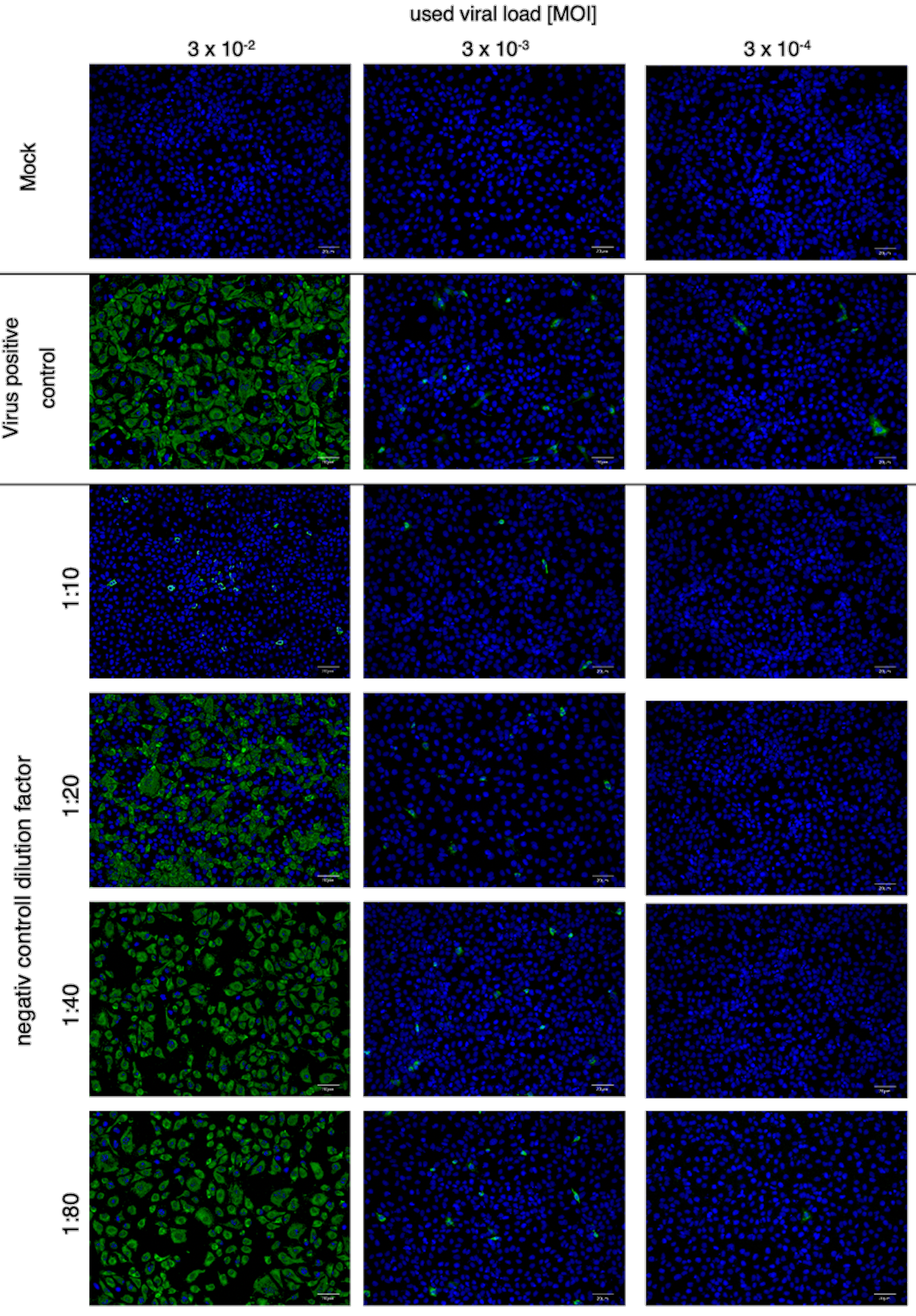

Supplement: Supplementary Material 1 — Detailed final protocol. [file Image1.tiff]

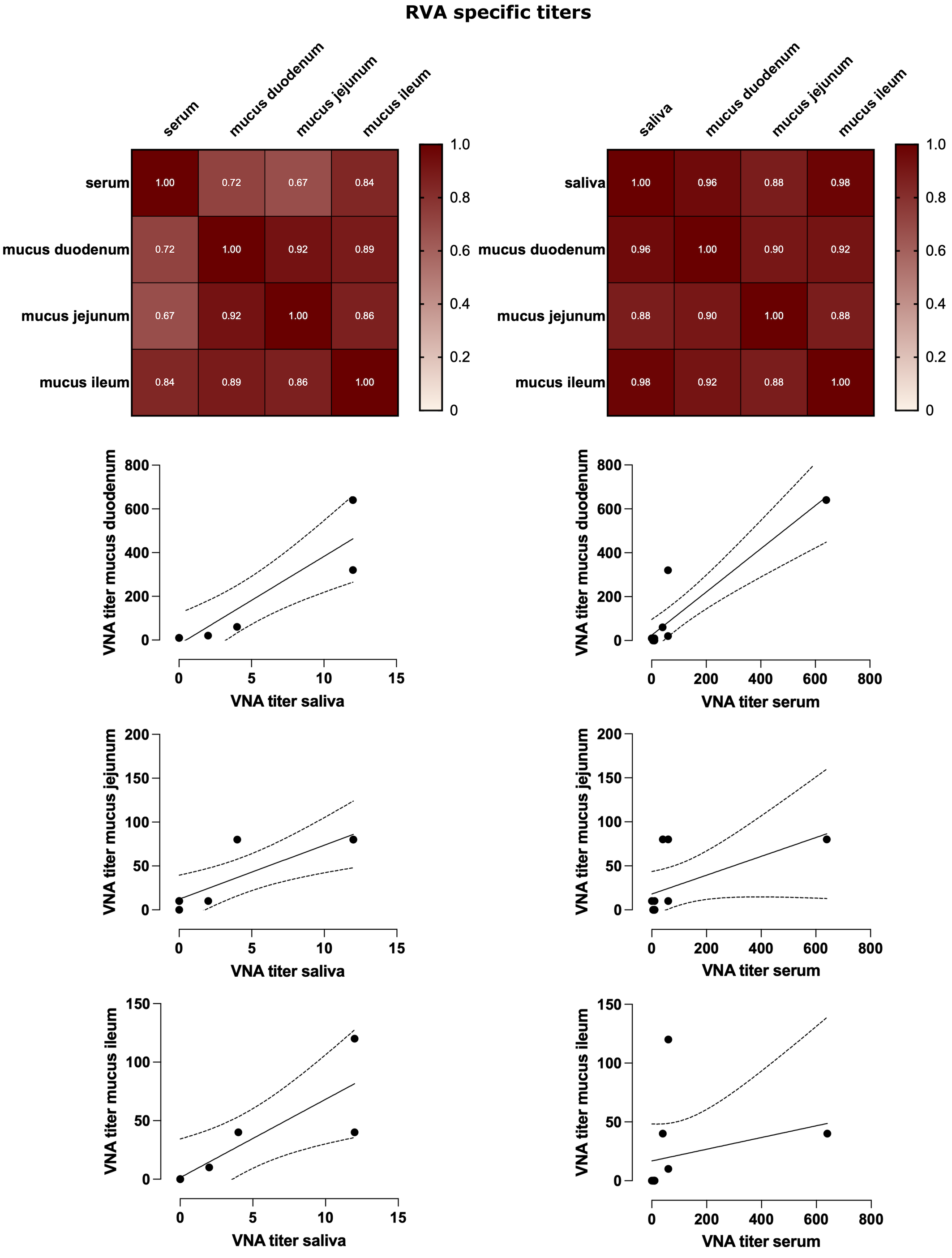

Supplement: Supplementary Material 2 — Determination of the Optimal Multiplicity of Infection (MOI). Virus loads corresponding to MOIs of 3 × 10^-2^ (left), 3 × 10^-3^ (center), and 3 × 10^-4^ (right) were used in the virus neutralization assay (VNA). As the serum negative controll was progressively diluted, the number of foci per well increased accordingly. At MOIs of 3 × 10^-2^ and 3 × 10^-3^, serum negative controll showed a VNA titer < 10. In contrast, at an MOI of 3 × 10^-4^, the VNA titer increased to 40. [file Image2.tiff]
