# Supplementary material for: Rotavirus-specific neutralization assay enables evaluation of mucosal immune responses
Source: Front Immunol. 2025 Dec 2;16:1677823. doi: 10.3389/fimmu.2025.1677823 (PMC12706807; doi:10.3389/fimmu.2025.1677823)
Supplement: Supplementary Material 3 — Correlation calculations between RVA-specific VNA titers within sample materials. The correlations between the intestinal mucus VNA titers and the saliva VNA titers or the serum VNA titers are shown graphically in the form of XY plots and a heat map. A correlation line is shown for correlations ≥ 0.5 (Spearman correlation, n = 5 or 7). [file DataSheet1.pdf]

Supplementary Material 1

Detailed Protocol of the Virus Neutralization Assay

---

1. Materials

(See main manuscript.)

---

2. Sampling of Material

Saliva

- Saliva is collected using a cotton roll (Salivette®) attached to a rod.
- Animals are allowed to chew on the cotton roll for approximately 20 s.
- The cotton roll is then placed into the centrifugation tube.

Blood

- Large blood vessels near the heart are incised, and blood is collected into serum tubes (~5 cm from the incision site).

Intestinal mucus

- The intestine is excised, separated from the mesentery, and flushed with hand-warm water.
- The lumen is everted and washed again with hand-warm water.
- Mucus is scraped using a blunt scalpel at three locations:
  - Duodenum (~10 cm distal to the gastric outlet)
  - Jejunum (midpoint)
  - Ileum (~10 cm proximal to the ileocecal junction)
- All samples are transferred into sterile 2 ml reaction tubes.

---

3. Sample Processing

- **Blood:** Samples are centrifuged at 2,000 × *g* for 5 min; serum is separated and stored at –80 °C.
- **Intestinal mucus:** Samples are diluted 1:50 in PBS, homogenized in a bead mill (30 s, 2,000 oscillations min^{-1}), and stored at –80 °C.
- **Saliva:** Samples are centrifuged at 2,000 × *g* for 5 min; the flow-through is collected in a 2 ml reaction tube, filtered through a 0.45 µm sterile filter, and stored at –80 °C.

---

4. MA104 Cell Culture

- MA104 cells are cultured in DMEM (with phenol red) supplemented with 10 % fetal calf serum at 37 °C, 5 % CO_2, and 90 % humidity.
- Cells are passaged twice weekly at 90–100 % confluence:
    - The medium is removed and cells are washed with PBS.
    - Cells are detached using 0.05 % trypsin–EDTA; detachment is monitored by phase-contrast microscopy.
    - For mucus samples, the reaction is stopped by adding DMEM containing 10 % fetal calf serum.
    - For serum and saliva samples, DMEM is added to the cell suspension, followed by centrifugation at 700 × *g* for 5 min. The pellet is resuspended in DMEM.

Determination of Cell Concentration

- 50 µl of the cell suspension are transferred to a 2 ml reaction tube.
- 10 µl trypan blue (1:6) are added to assess cell viability.
- Cells are counted using a Neubauer chamber and adjusted to 0.4×10^5 cells per well in 96-well plates.
- For mucus samples, cells are seeded in 96-well plates and the assay is continued the following day.
- For serum and saliva samples, cells are seeded in the virus–sample suspension after the designated adsorption time.

---

5. Virus Neutralization Assay (VNA)

5.1 Virus Preparation

- Virus stocks are adjusted with DMEM containing 20 µg ml^{-1} trypsin to the working dilution (MOI as indicated in Table 3).
- The virus is activated for 2 h at 37 °C.

5.2 Sample Dilution

- Two-fold serial dilutions are prepared in 96-well plates.
    - Serum: pre-diluted 1:10 (RVA) or 1:50 (RVC).
    - Saliva and mucus: used directly in the dilution series.
- 25 µl of pre-diluted samples are added to rows A–C.
- 25 µl DMEM are added from row H upwards to C in each well.
- The first dilution step (row C) is mixed by pipetting up and down 20 times.
- 25 µl are transferred successively from row C to row D and so on.
- 25 µl of activated virus are added to wells in rows B–H; row A receives 25 µl DMEM (negative control).
- Use two wells for the virus positive control. Therefore add 25 µl of virus suspension to a well and add another 75 µl of DMEM
- Plates are gently mixed and incubated as follows:
    - Serum: 2 h at 37 °C
    - Saliva and mucus: 8 h at 37 °C

5.3 Cell Addition and Incubation

- For serum and saliva: 4×10^4 MA104 cells per well in 50 µl are added to the virus–sample mixture.
- For intestinal mucus: 50 µl DMEM are added to the mixture, which is then transferred to confluent MA104 monolayers. The cell layer is washed with PBS before adding the virus–sample mixture. Plates are incubated for 2 h at 37 °C, 5 % CO_2, then washed twice with DMEM.
- Plates are incubated for 24 h at 37 °C, 5 % CO_2.

5.4 Fixation of the assay

- Cells are fixed with 80 % acetone at –20 °C for 15 min, washed twice with PBS, and stored at 4 °C.

---

6. Immunofluorescence Assay (IFA)

- Primary antibodies are added and incubated for 1 h at 37 °C:
  - Rabbit anti-RVA VP6 (1:2,000 in PBS + 1 % BSA)
  - Rabbit anti-RVC VP6 (1:4,000 in PBS + 1 % BSA)
- Wells are washed three times with PBS + 1 % BSA.
- Secondary antibody incubation:
  - Goat anti-rabbit IgG (H+L) Alexa Fluor™ 488 (1 $\mu g\ ml^{-1}$ in PBS + 1 % BSA)
  - DAPI (0.5 $\mu g\ ml^{-1}$)
  - Incubate for 30 min at 37 °C.
- Wells are washed twice with PBS + 1 % BSA and once with PBS.

---

7. Readout

- VP6-specific fluorescence signals are visualized using an inverted fluorescence microscope.
- The VNA titer is defined as the reciprocal of the highest sample dilution showing no specific fluorescence.
- If duplicates differ by one dilution step, the mean titer is reported.
- If duplicates differ by more than one dilution step, the assay is repeated.
